# The genome sequence of the White-shouldered Marble, *Apotomis turbidana* (Hübner, 1825)

**DOI:** 10.12688/wellcomeopenres.19242.1

**Published:** 2023-03-22

**Authors:** Douglas Boyes, James Hammond, University of Oxford and Wytham Woods Genome Acquisition Lab, Darwin Tree of Life Barcoding collective, Wellcome Sanger Institute Tree of Life programme, Wellcome Sanger Institute Scientific Operations: DNA Pipelines collective, Tree of Life Core Informatics collective, Darwin Tree of Life Consortium

**Affiliations:** 1 UK Centre for Ecology & Hydrology, Wallingford, England, UK; 2 University of Oxford, Oxford, England, UK

**Keywords:** Apotomis turbidana, White-shouldered Marble, genome sequence, chromosomal, Lepidoptera

## Abstract

We present a genome assembly from an individual male *Apotomis turbidana* (the White-shouldered Marble; Arthropoda; Insecta; Lepidoptera; Tortricidae). The genome sequence is 720.5 megabases in span. Most of the assembly is scaffolded into 28 chromosomal pseudomolecules, including the assembled Z sex chromosome. The mitochondrial genome has also been assembled and is 16.8 kilobases in length. Gene annotation of this assembly on Ensembl identified 22,646 protein coding genes.

## Species taxonomy

Eukaryota; Metazoa; Ecdysozoa; Arthropoda; Hexapoda; Insecta; Pterygota; Neoptera; Endopterygota; Lepidoptera; Glossata; Ditrysia; Tortricoidea; Tortricidae; Olethreutinae; Olethreutini; *Apotomis*; *Apotomis turbidana* (Hübner, 1825) (NCBI:txid 1100916).

## Background

*Apotomis turbidana* (Hübner, 1825) is a moth of the Tortricidae family. *A. turbidana* is one of many moths in its family which exhibit cryptic colouration, resembling bird droppings, a polyphyletic group known as the ‘bird-dropping tortricids’. The forewing markings are a mixture of white and charcoal-grey, and the species exhibits only minor variation in this ( Bradley *et al.*, 1979). There is, however, a distinct form from the west of Ireland with silver strigulations and a brownish colour in place of the charcoal-grey markings ( Bradley *et al.*, 1979).

Larvae feed on birch ( *Betula*) between April and May, between spun leaves ( Bradley *et al.*, 1979; Elliott *et al.*, 2018). Larvae have also been recorded feeding on *Salix*, *Populus*, and *Quercus*, elsewhere in Europe ( Bradley *et al.*, 1979; Hancock *et al.*, 2015). Pupation occurs between spun leaves or within the larval habitation in June, and adult moths can be found between June and July ( Bradley *et al.* 1979; Elliott *et al.*, 2018; Hancock *et al.*, 2015). Adults have been found in August and September, suggesting a possible second generation ( Elliott *et al.*, 2018). Adult moths fly from before dusk and come to light ( Bradley *et al.*, 1979; Elliott *et al.*, 2018; Hancock *et al.*, 2015).

The moth is widespread across Great Britain and Ireland, found in habitats with birch woodland ( Bradley *et al.*, 1979). Globally the species is found in northern and central Europe, ranging east to Siberia ( Bradley *et al.*, 1979; GBIF Secretariat, 2022; Hancock *et al.*, 2015).

A genome of *Apotomis turbidana* will facilitate research into the evolution of cryptic colouration in lepidoptera, and its genomic basis. The genome of *A. turbidana* was sequenced as part of the Darwin Tree of Life Project, a collaborative effort to sequence all named eukaryotic species in the Atlantic Archipelago of Britain and Ireland. Here we present a chromosomally complete genome sequence for *A. turbidana*, based on one male specimen from Wytham Woods, Oxfordshire, UK.

### Genome sequence report

**Figure 1.**
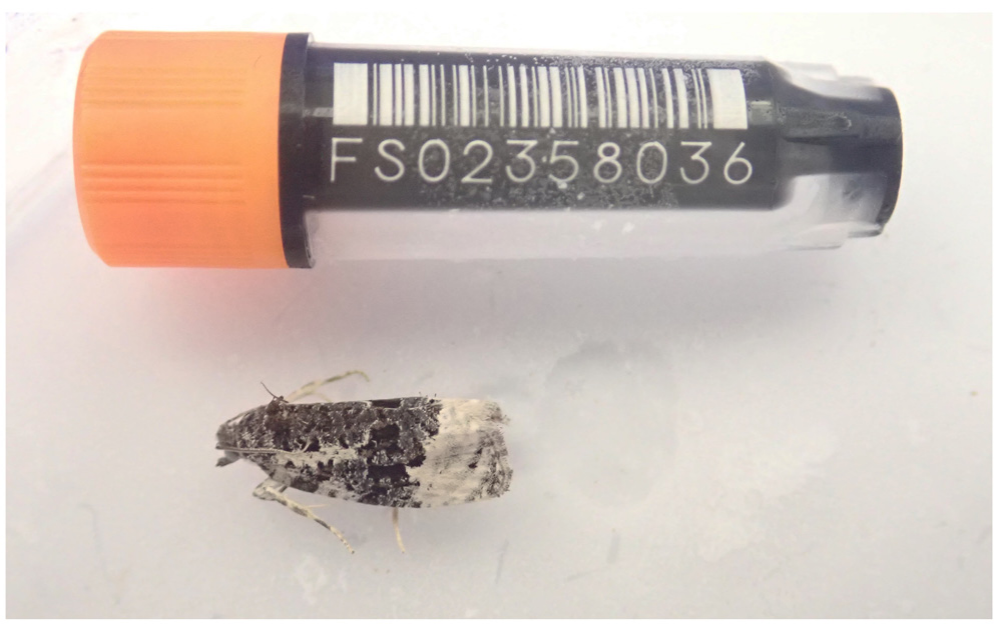
Photograph of the *Apotomis turbidana* (ilApoTurb1) specimen used for genome sequencing.

The genome was sequenced from one male *Apotomis turbidana* specimen ( Figure 1) collected from Wytham Woods, Oxfordshire, UK (latitude 51.77, longitude –1.34). A total of 32-fold coverage in Pacific Biosciences single-molecule HiFi long reads and 52-fold coverage in 10X Genomics read clouds was generated. Primary assembly contigs were scaffolded with chromosome conformation Hi-C data. Manual assembly curation corrected 75 missing or mis-joins and removed 22 haplotypic duplications, reducing the assembly length by 1.35% and the scaffold number by 48.81%, and increasing the scaffold N50 by 9.16%.

**Figure 2.**
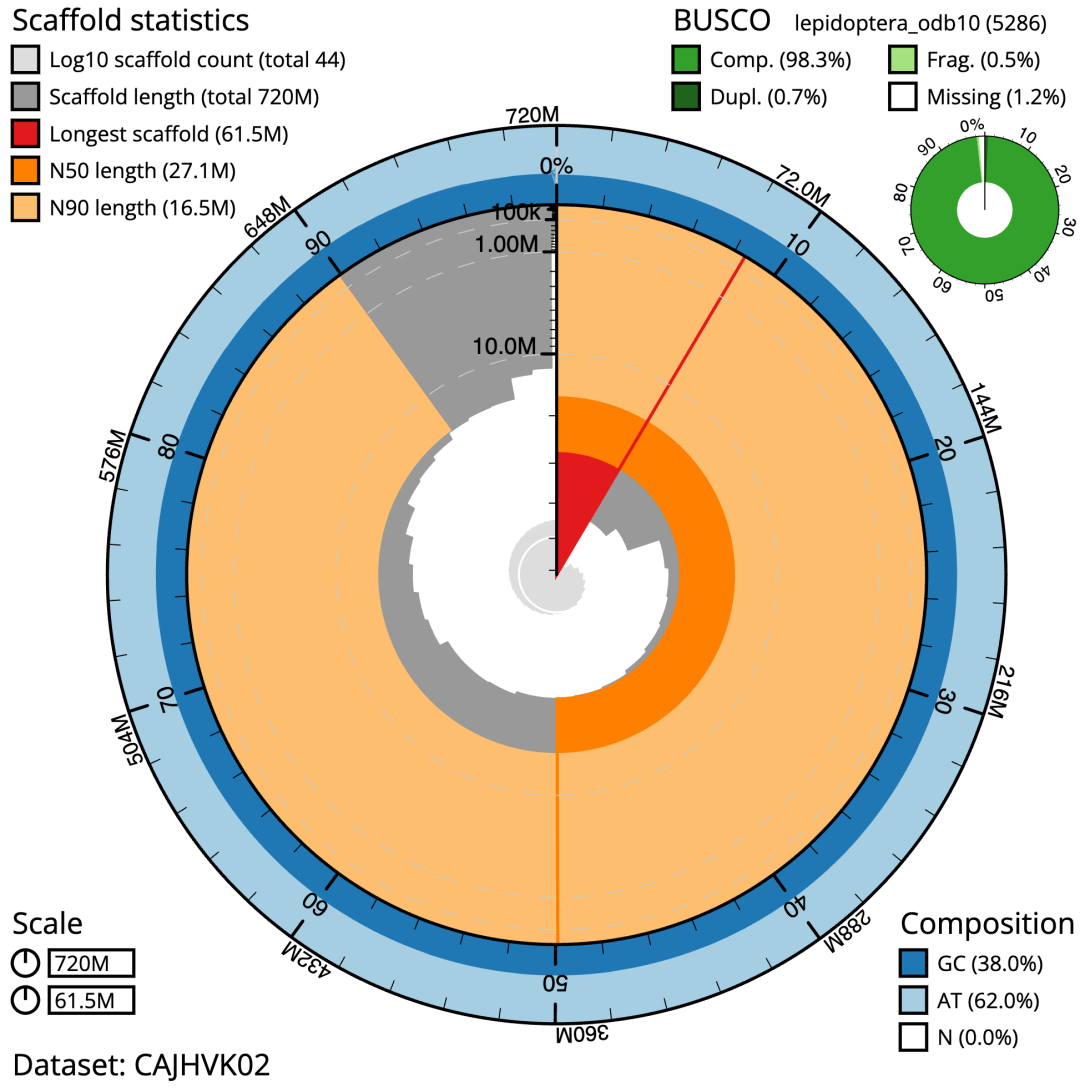
Genome assembly of *Apotomis turbidana*, ilApoTurb1.2: metrics. The BlobToolKit Snailplot shows N50 metrics and BUSCO gene completeness. The main plot is divided into 1,000 size-ordered bins around the circumference with each bin representing 0.1% of the 720,467,098 bp assembly. The distribution of scaffold lengths is shown in dark grey with the plot radius scaled to the longest scaffold present in the assembly (61,450,864 bp, shown in red). Orange and pale-orange arcs show the N50 and N90 scaffold lengths (27,109,628 and 16,472,394 bp), respectively. The pale grey spiral shows the cumulative scaffold count on a log scale with white scale lines showing successive orders of magnitude. The blue and pale-blue area around the outside of the plot shows the distribution of GC, AT and N percentages in the same bins as the inner plot. A summary of complete, fragmented, duplicated and missing BUSCO genes in the lepidoptera_odb10 set is shown in the top right. An interactive version of this figure is available at https://blobtoolkit.genomehubs.org/view/ilApoTurb1.2/dataset/CAJHVK02/snail.

The final assembly has a total length of 720.5 Mb in 43 sequence scaffolds with a scaffold N50 of 27.1 Mb ( Table 1). Most (99.98%) of the assembly sequence was assigned to 28 chromosomal-level scaffolds, representing 27 autosomes, and the Z sex chromosome. Chromosome-scale scaffolds confirmed by the Hi-C data are named in order of size ( Figure 2– Figure 5; Table 2). While not fully phased, the assembly deposited is of one haplotype. Contigs corresponding to the second haplotype have also been deposited.

**Table 1.** Genome data for *Apotomis turbidana*, ilApoTurb1.2.

Project accession data		
Assembly identifier	ilApoTurb1.2	
Species	*Apotomis turbidana*	
Specimen	ilApoTurb1	
NCBI taxonomy ID	1100916	
BioProject	PRJEB42113	
BioSample ID	SAMEA7520681	
Isolate information	ilApoTurb1, male, whole organism (genome sequencing and Hi-C scaffolding)	
Assembly metrics *		*Benchmark*
Consensus quality (QV)	55.7	*≥ 50*
*k*-mer completeness	99.99%	*≥ 95%*
BUSCO **	C:98.3%[S:97.6%,D:0.7%], F:0.5%,M:1.2%,n:5,286	*C ≥ 95%*
Percentage of assembly mapped to chromosomes	99.98%	*≥ 95%*
Sex chromosomes	Z chromosome	*localised* *homologous pairs*
Organelles	Mitochondrial genome assembled	*complete single* *alleles*
Raw data accessions		
PacificBiosciences SEQUEL II	ERR6436363	
10X Genomics Illumina	ERR6002581–ERR6002584	
Hi-C Illumina	ERR6002585	
Genome assembly		
Assembly accession	GCA_905147355.2	
*Accession of alternate haplotype*	GCA_905147195.1	
Span (Mb)	720.5	
Number of contigs	109	
Contig N50 length (Mb)	13.8	
Number of scaffolds	43	
Scaffold N50 length (Mb)	27.1	
Longest scaffold (Mb)	61.5	
Genome annotation		
Number of protein-coding genes	22,646	
Number of non-coding genes	22,880	

* Assembly metric benchmarks are adapted from column VGP-2020 of “Table 1: Proposed standards and metrics for defining genome assembly quality” from ( Rhie *et al.*, 2021). ** BUSCO scores based on the lepidoptera_odb10 BUSCO set using v5.3.2. C = complete [S = single copy, D = duplicated], F = fragmented, M = missing, n = number of orthologues in comparison. A full set of BUSCO scores is available at https://blobtoolkit.genomehubs.org/view/ilApoTurb1.2/dataset/CAJHVK02/busco.

**Figure 3.**
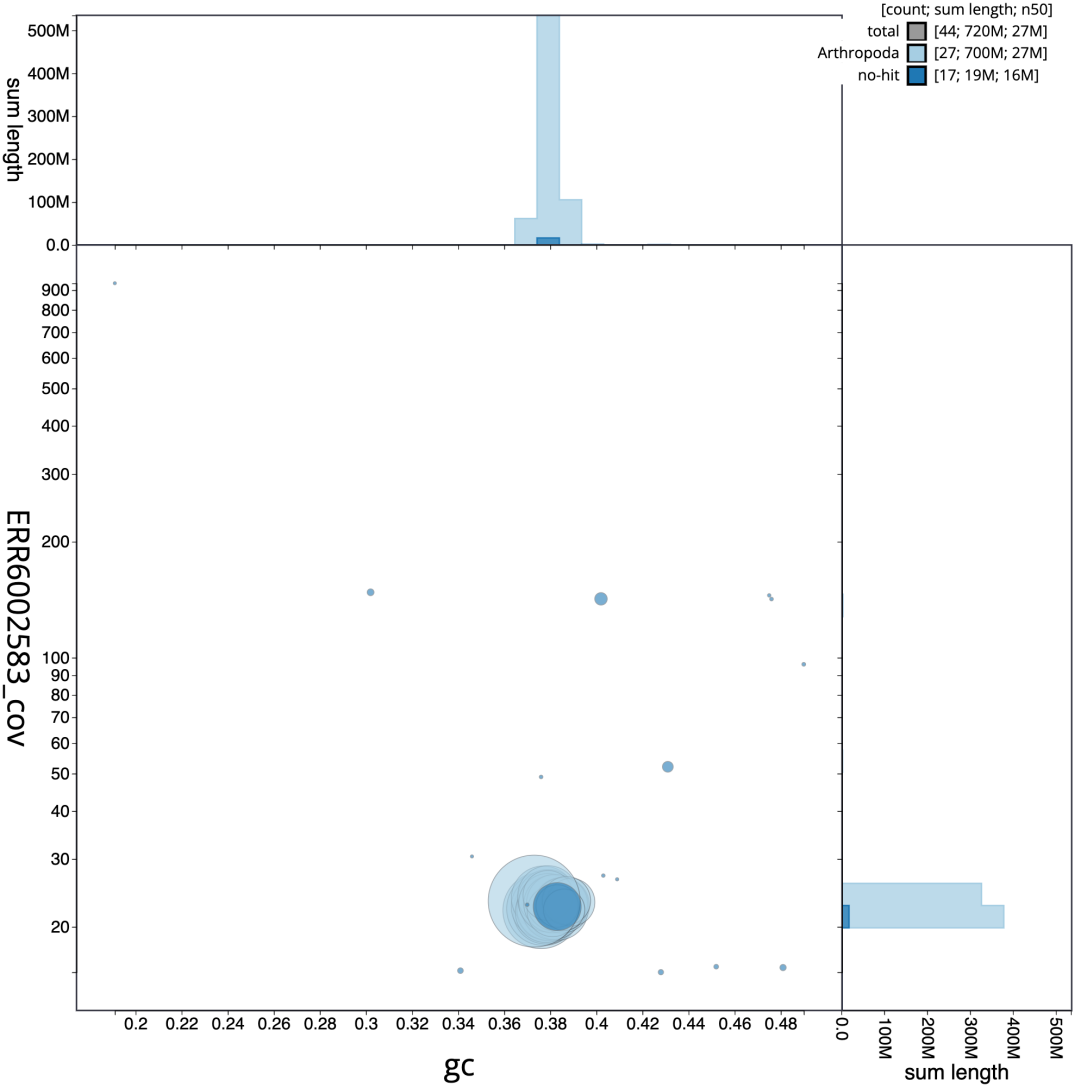
Genome assembly of *Apotomis turbidana*, ilApoTurb1.2: GC coverage. BlobToolKit GC-coverage plot. Scaffolds are coloured by phylum. Circles are sized in proportion to scaffold length. Histograms show the distribution of scaffold length sum along each axis. An interactive version of this figure is available at https://blobtoolkit.genomehubs.org/view/ilApoTurb1.2/dataset/CAJHVK02/blob.

**Figure 4.**
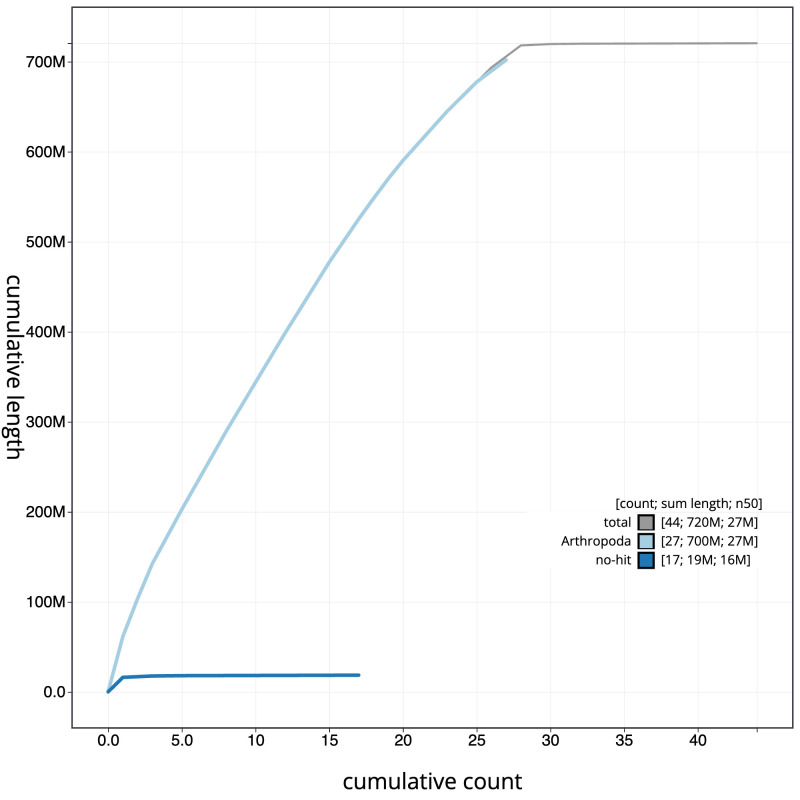
Genome assembly of *Apotomis turbidana*, ilApoTurb1.2: cumulative sequence. BlobToolKit cumulative sequence plot. The grey line shows cumulative length for all scaffolds. Coloured lines show cumulative lengths of scaffolds assigned to each phylum using the buscogenes taxrule. An interactive version of this figure is available at https://blobtoolkit.genomehubs.org/view/ilApoTurb1.2/dataset/CAJHVK02/cumulative.

**Figure 5.**
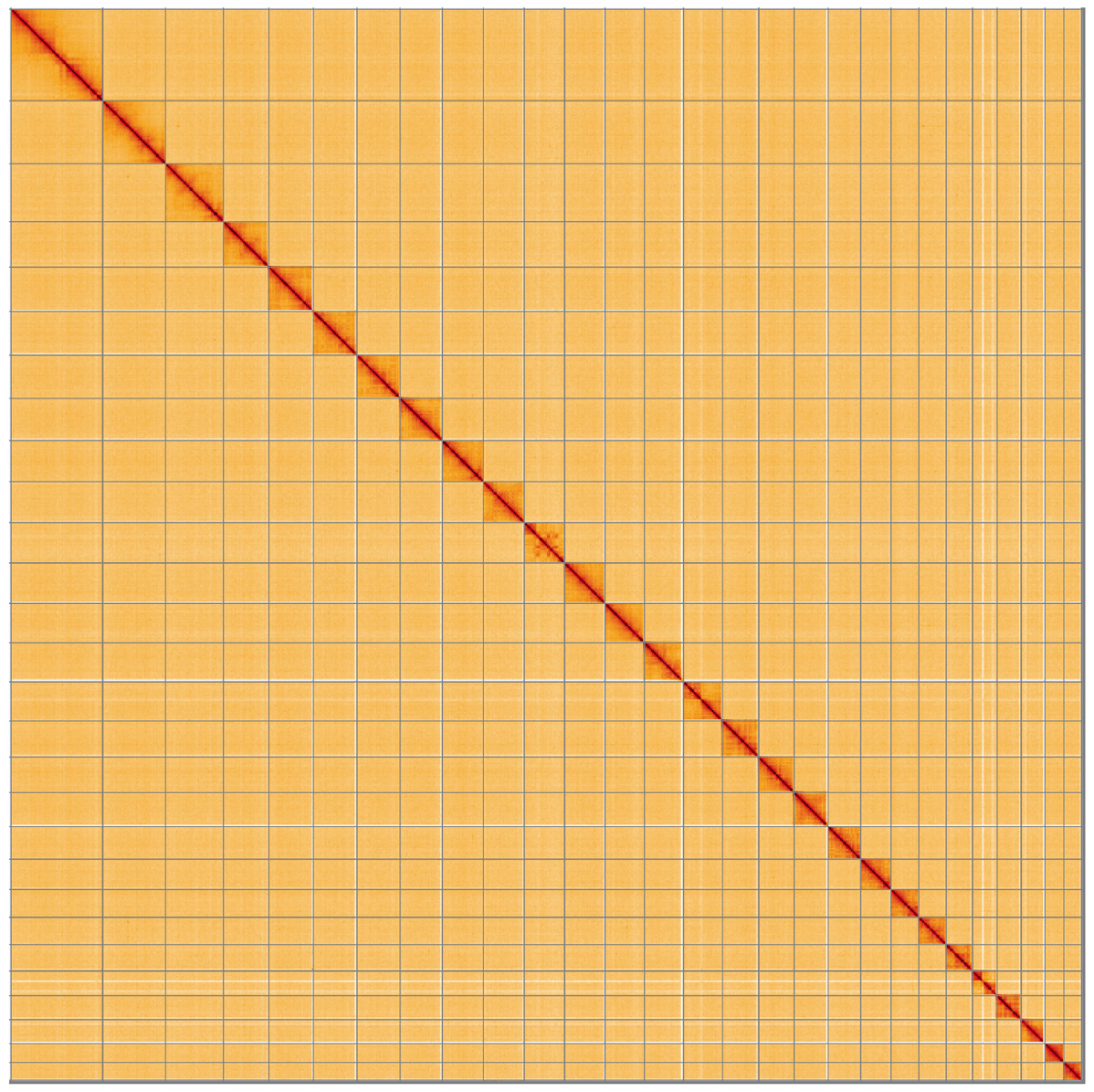
Genome assembly of *Apotomis turbidana*, ilApoTurb1.2: Hi-C contact map. Hi-C contact map of the ilApoTurb1.2 assembly, visualised using HiGlass. Chromosomes are shown in order of size from left to right and top to bottom. An interactive version of this figure may be viewed at https://genome-note-higlass.tol.sanger.ac.uk/l/?d=Yp8m4GnjTNyRY1bT4meMEw.

**Table 2.** Chromosomal pseudomolecules in the genome assembly of *Apotomis turbidana*, ilApoTurb1.

INSDC accession	Chromosome	Size (Mb)	GC%
LR990281.1	1	42.16	37.6
LR990282.1	2	38.83	37.7
LR990283.1	3	30.53	37.8
LR990284.1	4	29.68	37.7
LR990285.1	5	29.32	37.7
LR990286.1	6	28.9	37.9
LR990287.1	7	28.33	38.1
LR990289.1	8	27.34	37.9
LR990288.1	9	27.59	38.1
LR990290.1	10	27.11	38.3
LR990291.1	11	27.11	38.1
LR990292.1	12	26.43	37.8
LR990293.1	13	26.21	37.9
LR990294.1	14	26.17	37.9
LR990295.1	15	24.32	37.9
LR990296.1	16	23.72	38.1
LR990297.1	17	22.75	38.2
LR990298.1	18	21.91	38.5
LR990299.1	19	20.31	38.5
LR990300.1	20	18.67	38.5
LR990301.1	21	18.36	38.1
LR990302.1	22	17.69	37.9
LR990305.1	23	16.01	38.9
LR990303.1	24	16.47	38.7
LR990304.1	25	16.13	38.3
LR990306.1	26	12.61	38.3
LR990307.1	27	11.91	38.6
LR990280.1	Z	61.45	37.3
LR990308.2	MT	0.02	19.1
-	unplaced	2.41	40.8

The estimated *k*-mer-based Quality Value (QV) of the final assembly is 55.7 with *k*-mer based completeness of 99.99%, and the assembly has a BUSCO v5.3.2 ( Manni *et al.*, 2021) completeness of 98.3% (single 97.6%, duplicated 0.7%) using the lepidoptera_odb10 reference set ( *n* = 5,286).

### Genome annotation report

The *A. turbidana* genome assembly GCA_905147355.2 was annotated using the Ensembl rapid annotation pipeline ( Table 1; https://rapid.ensembl.org/Apotomis_turbidana_GCA_905147355.2/Info/Index/). The resulting annotation includes 22,880 transcribed mRNAs from 22,646 protein-coding genes.

## Methods

### Sample acquisition and nucleic acid extraction

A male *Apotomis turbidana* specimen (ilApoTurb1) was collected from Wytham Woods, Oxfordshire (biological vice-county: Berkshire), UK (latitude 51.77, longitude –1.34) on 13 June 2020. The specimen was taken from woodland habitat by Douglas Boyes (University of Oxford) using a light trap. The specimen was identified by the collector and snap-frozen on dry ice.

DNA was extracted at the Tree of Life laboratory, Wellcome Sanger Institute (WSI). The ilApoTurb1 sample was weighed and dissected on dry ice with tissue set aside for Hi-C sequencing. Whole organism tissue was disrupted using a Nippi Powermasher fitted with a BioMasher pestle. High molecular weight (HMW) DNA was extracted using the Qiagen MagAttract HMW DNA extraction kit. Low molecular weight DNA was removed from a 20 ng aliquot of extracted DNA using the 0.8X AMpure XP purification kit prior to 10X Chromium sequencing; a minimum of 50 ng DNA was submitted for 10X sequencing. HMW DNA was sheared into an average fragment size of 12–20 kb in a Megaruptor 3 system with speed setting 30. Sheared DNA was purified by solid-phase reversible immobilisation using AMPure PB beads with a 1.8X ratio of beads to sample to remove the shorter fragments and concentrate the DNA sample. The concentration of the sheared and purified DNA was assessed using a Nanodrop spectrophotometer and Qubit Fluorometer and Qubit dsDNA High Sensitivity Assay kit. Fragment size distribution was evaluated by running the sample on the FemtoPulse system.

### Sequencing

Pacific Biosciences HiFi circular consensus and 10X Genomics read cloud DNA sequencing libraries were constructed according to the manufacturers’ instructions. DNA sequencing was performed by the Scientific Operations core at the WSI on Pacific Biosciences SEQUEL II (HiFi) and HiSeq X Ten (10X) instruments. Hi-C data were also generated from tissue of ilApoTurb1 using the Arima v2 kit and sequenced on the HiSeq X Ten instrument.

### Genome assembly, curation and evaluation

Assembly was carried out with Hifiasm ( Cheng *et al.*, 2021) and haplotypic duplication was identified and removed with purge_dups ( Guan *et al.*, 2020). One round of polishing was performed by aligning 10X Genomics read data to the assembly with Long Ranger ALIGN, calling variants with FreeBayes ( Garrison & Marth, 2012). The assembly was then scaffolded with Hi-C data ( Rao *et al.*, 2014) using SALSA2 ( Ghurye *et al.*, 2019). The assembly was checked for contamination and corrected using the gEVAL system ( Chow *et al.*, 2016) as described previously ( Howe *et al.*, 2021). Manual curation was performed using gEVAL, HiGlass ( Kerpedjiev *et al.*, 2018) and Pretext ( Harry, 2022). The mitochondrial genome was assembled using MitoHiFi ( Uliano-Silva *et al.*, 2022), which performed annotation using MitoFinder ( Allio *et al.*, 2020). To evaluate the assembly, MerquryFK was used to estimate consensus quality (QV) scores and *k*-mer completeness ( Rhie *et al.*, 2020). The genome was analysed and BUSCO scores ( Manni *et al.*, 2021; Simão *et al.*, 2015) were generated within the BlobToolKit environment ( Challis *et al.*, 2020). Table 3 contains a list of software tool versions and sources.

**Table 3.** Software tools: versions and sources.

Software tool	Version	Source
BlobToolKit	4.0.7	https://github.com/blobtoolkit/ blobtoolkit
BUSCO	5.3.2	https://gitlab.com/ezlab/busco
FreeBayes	1.3.1-17- gaa2ace8	https://github.com/freebayes/ freebayes
gEVAL	N/A	https://geval.org.uk/
Hifiasm	0.12	https://github.com/chhylp123/ hifiasm
HiGlass	1.11.6	https://github.com/higlass/higlass
Long Ranger ALIGN	2.2.2	https://support.10xgenomics.com/ genome-exome/software/pipelines/ latest/advanced/other-pipelines
Merqury	MerquryFK	https://github.com/thegenemyers/ MERQURY.FK
MitoHiFi	1	https://github.com/marcelauliano/ MitoHiFi
PretextView	0.2	https://github.com/wtsi-hpag/ PretextView
purge_dups	1.2.3	https://github.com/dfguan/purge_ dups
SALSA	2.2	https://github.com/salsa-rs/salsa

### Genome annotation

The BRAKER2 pipeline ( Brůna *et al.*, 2021) was used in the default protein mode to generate annotation for the *Apotomis turbidana* assembly (GCA_905147355.2). in Ensembl Rapid Release.

### Ethics and compliance issues

The materials that have contributed to this genome note have been supplied by a Darwin Tree of Life Partner. The submission of materials by a Darwin Tree of Life Partner is subject to the Darwin Tree of Life Project Sampling Code of Practice. By agreeing with and signing up to the Sampling Code of Practice, the Darwin Tree of Life Partner agrees they will meet the legal and ethical requirements and standards set out within this document in respect of all samples acquired for, and supplied to, the Darwin Tree of Life Project. All efforts are undertaken to minimise the suffering of animals used for sequencing. Each transfer of samples is further undertaken according to a Research Collaboration Agreement or Material Transfer Agreement entered into by the Darwin Tree of Life Partner, Genome Research Limited (operating as the Wellcome Sanger Institute), and in some circumstances other Darwin Tree of Life collaborators.

## Data Availability

European Nucleotide Archive: *Apotomis turbidana* (white-shouldered marble). Accession number PRJEB42113; https://identifiers.org/ena.embl/PRJEB42113. ( Wellcome Sanger Institute, 2022) The genome sequence is released openly for reuse. The *Apotomis turbidana* genome sequencing initiative is part of the Darwin Tree of Life (DToL) project. All raw sequence data and the assembly have been deposited in INSDC databases. Raw data and assembly accession identifiers are reported in Table 1.

## Author information

Members of the University of Oxford and Wytham Woods Genome Acquisition Lab are listed here: https://doi.org/10.5281/zenodo.4789928.

Members of the Darwin Tree of Life Barcoding collective are listed here: https://doi.org/10.5281/zenodo.4893703.

Members of the Wellcome Sanger Institute Tree of Life programme are listed here: https://doi.org/10.5281/zenodo.4783585.

Members of Wellcome Sanger Institute Scientific Operations: DNA Pipelines collective are listed here: https://doi.org/10.5281/zenodo.4790455.

Members of the Tree of Life Core Informatics collective are listed here: https://doi.org/10.5281/zenodo.5013541.

Members of the Darwin Tree of Life Consortium are listed here: https://doi.org/10.5281/zenodo.4783558.

## References

[ref-1] Allio R Schomaker-Bastos A Romiguier J : MitoFinder: Efficient automated large‐scale extraction of mitogenomic data in target enrichment phylogenomics. *Mol Ecol Resour.* 2020;20(4):892–905. 10.1111/1755-0998.13160 32243090 PMC7497042

[ref-2] Bradley J Tremewan WG Smith A : British Tortricoid Moths - Tortricidae: Olethreutinae.The Ray Society,1979.

[ref-3] Brůna T Hoff KJ Lomsadze A : BRAKER2: Automatic eukaryotic genome annotation with GeneMark-EP+ and AUGUSTUS supported by a protein database. *NAR Genom Bioinform.* 2021;3(1):lqaa108. 10.1093/nargab/lqaa108 33575650 PMC7787252

[ref-4] Challis R Richards E Rajan J : BlobToolKit - interactive quality assessment of genome assemblies. *G3 (Bethesda).* 2020;10(4):1361–1374. 10.1534/g3.119.400908 32071071 PMC7144090

[ref-5] Cheng H Concepcion GT Feng X : Haplotype-resolved *de novo* assembly using phased assembly graphs with hifiasm. *Nat Methods.* 2021;18(2):170–175. 10.1038/s41592-020-01056-5 33526886 PMC7961889

[ref-6] Chow W Brugger K Caccamo M : gEVAL - a web-based browser for evaluating genome assemblies. *Bioinformatics.* 2016;32(16):2508–10. 10.1093/bioinformatics/btw159 27153597 PMC4978925

[ref-7] Elliott B : Tortricidae.In: J.R. Langmaid, S.M. Palmer, and M.R. Young (eds) *A Field Guide to the Smaller Moths of Great Britain and Ireland.* The British Entomological and Natural History Society, 2018;279.

[ref-8] Garrison E Marth G : Haplotype-based variant detection from short-read sequencing. 2012. 10.48550/arXiv.1207.3907

[ref-9] GBIF Secretariat: Apotomis turbidana Hübner, 1825, GBIF Backbone Taxonomy. Checklist dataset. 2022; (Accessed: 9 March 2023). 10.15468/39omei

[ref-10] Ghurye J Rhie A Walenz BP : Integrating Hi-C links with assembly graphs for chromosome-scale assembly. *PLoS Comput Biol.* 2019;15(8):e1007273. 10.1371/journal.pcbi.1007273 31433799 PMC6719893

[ref-11] Guan D McCarthy SA Wood J : Identifying and removing haplotypic duplication in primary genome assemblies. *Bioinformatics.* 2020;36(9):2896–2898. 10.1093/bioinformatics/btaa025 31971576 PMC7203741

[ref-12] Hancock F Bland KP Razowski J : The Moths and Butterflies of Great Britain and Ireland.Leiden: BRILL,2015;5(2). Reference Source

[ref-13] Harry E : PretextView (Paired REad TEXTure Viewer): A desktop application for viewing pretext contact maps. 2022; (Accessed: 19 October 2022). Reference Source

[ref-14] Howe K Chow W Collins J : Significantly improving the quality of genome assemblies through curation. *GigaScience.* Oxford University Press,2021;10(1):giaa153. 10.1093/gigascience/giaa153 33420778 PMC7794651

[ref-15] Kerpedjiev P Abdennur N Lekschas F : HiGlass: Web-based visual exploration and analysis of genome interaction maps. *Genome Biol.* 2018;19(1):125. 10.1186/s13059-018-1486-1 30143029 PMC6109259

[ref-16] Manni M Berkeley MR Seppey M : BUSCO Update: Novel and Streamlined Workflows along with Broader and Deeper Phylogenetic Coverage for Scoring of Eukaryotic, Prokaryotic, and Viral Genomes. *Mol Biol Evol.* 2021;38(10):4647–4654. 10.1093/molbev/msab199 34320186 PMC8476166

[ref-17] Rao SSP Huntley MH Durand NC : A 3D map of the human genome at kilobase resolution reveals principles of chromatin looping. *Cell.* 2014;159(7):1665–80. 10.1016/j.cell.2014.11.021 25497547 PMC5635824

[ref-19] Rhie A McCarthy SA Fedrigo O : Towards complete and error-free genome assemblies of all vertebrate species. *Nature.* 2021;592(7856):737–746. 10.1038/s41586-021-03451-0 33911273 PMC8081667

[ref-18] Rhie A Walenz BP Koren S : Merqury: Reference-free quality, completeness, and phasing assessment for genome assemblies. *Genome Biol.* 2020;21(1):245. 10.1186/s13059-020-02134-9 32928274 PMC7488777

[ref-20] Simão FA Waterhouse RM Ioannidis P : BUSCO: assessing genome assembly and annotation completeness with single-copy orthologs. *Bioinformatics.* 2015;31(19):3210–2. 10.1093/bioinformatics/btv351 26059717

[ref-21] Uliano-Silva M Ferreira JGRN Krasheninnikova K : MitoHiFi: a python pipeline for mitochondrial genome assembly from PacBio High Fidelity reads. *bioRxiv.* [Preprint],2022. 10.1101/2022.12.23.521667 PMC10354987 37464285

[ref-22] Wellcome Sanger Institute: The genome sequence of the White-shouldered marble, *Apotomis turbidana* (Hübner, 1825). European Nucleotide Archive.[dataset], accession number PRJEB42113,2022.

