# Supplementary material for: The genome sequence of the White-shouldered Marble, Apotomis turbidana (Hübner, 1825)
Source: Wellcome Open Res. 2023 Mar 22;8:127. [Version 1] doi: 10.12688/wellcomeopenres.19242.1 (PMC10442595; doi:10.12688/wellcomeopenres.19242.1)
Supplement: Peer review history

# Peer review: Reviewer response for version 1

**DOI:** 10.21956/wellcomeopenres.21325.r65094

**Published:** 2023-08-21

**Authors:** Annabel Whibley

**Affiliations:** 1 The University of Auckland, Auckland, Auckland, New Zealand; 2 Bragato Research Institute, Riversdale, New Zealand

This data note presents high-quality, foundational genomic resources for the tortrid moth species Apotomis turbidana (commonly known as the White-shouldered Marble). The report is extremely clearly set out, and uses appropriate and cutting-edge tools to support the construction, analysis, annotation and evaluation of the target genome. The quality of the assembly is excellent and the compilation and presentation of key statistics is clear and makes information and genome resources very easily available to the research community. The information provided in the natural history section of the introduction, whilst concise, is nonetheless comprehensive, relevant and engaging. The sole error I identified is typographical and minor- a stray “.” in the genome annotation methods section after the accession number.

Are sufficient details of methods and materials provided to allow replication by others?

Yes

Is the rationale for creating the dataset(s) clearly described?

Yes

Are the datasets clearly presented in a useable and accessible format?

Yes

Are the protocols appropriate and is the work technically sound?

Yes

Reviewer Expertise:

Genome biology and evolution

I confirm that I have read this submission and believe that I have an appropriate level of expertise to confirm that it is of an acceptable scientific standard.

# Peer review: Reviewer response for version 1

**DOI:** 10.21956/wellcomeopenres.21325.r63258

**Published:** 2023-08-17

**Authors:** Sara J. Oppenheim

**Affiliations:** 1 Sackler Institute for Comparative Genomics, American Museum of Natural History, New York, NY, USA

This report presents the sequencing, assembly , assembly and initial annotation of the genome of a charismatic moth, the bird poop moth. This is a solid project and the detailed presentation of assembly statistics and the well documented methods are appropriate and sufficient. The results are commendable and should be of use to the broader Lepidoptera research community.

Are sufficient details of methods and materials provided to allow replication by others?

Yes

Is the rationale for creating the dataset(s) clearly described?

Yes

Are the datasets clearly presented in a useable and accessible format?

Yes

Are the protocols appropriate and is the work technically sound?

Yes

Reviewer Expertise:

insect-plant interactions; insect ecology; insect genomics; comparative genomics; bioinformatics

I confirm that I have read this submission and believe that I have an appropriate level of expertise to confirm that it is of an acceptable scientific standard.
